# Editorial: Fungal diversity of forests: phylogeny, taxonomy and pathology

**DOI:** 10.3389/fmicb.2023.1353548

**Published:** 2024-01-16

**Authors:** Kezia Goldmann

**Affiliations:** Department Soil Ecology, UFZ-Helmholtz Centre for Environmental Research, Halle (Saale), Germany

**Keywords:** cultivation, high throughput sequencing, fungal ITS, DNA, trees, morphology, molecular tool box

Fungi represent a major proportion of the forest microbiome (Baldrian, 2017). Due to their taxonomic and functional diversity, nurtured by diverse forest habitats, fungi play an important role in maintaining the structure and functioning of these ecosystems (Mishra et al., 2020). Their enzymatic equipment enables saprotrophic fungi to break down recalcitrant organic matter, i.e., litter or deadwood – thus, released nutrients can be subsequently utilized by forest trees (Boddy and Watkinson, 1995). Mutualistic fungi can form close relationships with tree roots and facilitate their acquisition and uptake of nutrients (Smith and Read, 2010), and are essential for carbon sequestration (Clemmensen et al., 2015). Finally, pathogenic fungi, responsible for a wide range of tree diseases and even adaptations in forest management practices (Castello et al., 1995), form another fungal guild. Independent of fungal lifestyle, all niches given in a forest stand or even on a single plant are occupied by various fungal taxa (Baldrian, 2017).

Recent developments, the increased affordability and accessibility of high-throughput sequencing techniques coupled with optimized bioinformatics and phylogenetic analyses have enabled scientists to systematically explore fungal diversity, taxonomy, and evolution across scales. Advanced cultivation methods and tools for morphological trait determination can additionally support the functional characterization and identification of fungal pathogenicity.

Accordingly, this Research Topic aimed to present current research on fungal diversity within forests. Within this topic, five research articles were published which show the importance of fungi, especially in relation to their habitat or host plant.

Hofmann et al. displayed the importance of tree–fungus interactions by exploring the fungal communities of different tree niches. The authors aimed to gain understanding on how above- and belowground fungal communities are interconnected. Additionally, new insights in the scarcely studied fungi on the surfaces of tree bark could be gained. Hence, the bark surfaces of Norway spruce, Scots pine, and European beech and the soil in these respective forest plots were sampled in two temperate forests sites located in Germany. The resulting fungal communities, accessed using amplicon sequencing of the fungal internal transcribed spacer (ITS) region 2, appeared to be highly tree species- and tree niche-specific. This specificity was accompanied with adapted taxonomic patterns of the bark or soil inhabiting fungal communities. Only a few but common taxa were shared between either niche or tree species. The authors conclude that further research is needed to verify if the shared fungal taxa are environmental generalists or if they belong to the “core forest mycobiome.”

Douanla-Meli and Moll also studied the bark-associated fungal communities using ITS2 amplicon sequencing. However, their objective was to understand how the infection of European chestnut trees with different fungal pathogens overall impacts the bark-inhabiting community through monitoring and sampling different stages of the disease development, i.e., from infection to canker formation, and comparing this to bark samples from asymptomatic chestnut trees. Overall, the fungal communities of chestnut barks appeared very diverse and were dominated by Asco- and Basidiomycota. The formation of canker tissue, however, reduced the diversity of these fungi and led to shifts in taxonomic composition. As the presented study should be considered as a pilot, further investigations identifying bark-associated fungi in relation to tree diseases are needed. Additionally, cultivation and functional tests are requested to uncover natural antagonists to known fungal pathogens.

The study by Nascimento Brito et al. focused on the fungal genus *Trichoderma*, known for its ability to successfully control plant diseases, and its potential to serve as biocontrol. Through the combination of morphological observations with ITS amplicon sequencing and advanced phylogenetic analyses, the authors investigated 30 endophytic *Trichoderma* isolates obtained from the roots, stems, and leaves of rubber trees of the Brazilian Amazon. Even from this small sample size, numerous species of *Trichoderma*, of which four were described for the first time, could be identified and further characterized. Thus, the study confirms that disease management can be supported by resident fungal endophytes.

Another two publications nicely display that some research can only be obtained by the combination of sequencing techniques and detailed morphologic descriptions.

Hyphomycetes, commonly referred to as “fungi imperfecti”, are the focus of Lu et al. Fungi of this classification are taxonomically highly diverse but morphologically difficult to distinguish, which fascinates specialists. However, these fungi are able to produce secondary metabolites, potentially containing bioactive compounds, a circumstance which might lead to a broader interest in this fungal group. Yet, only by joining molecular and morphological analyses, the authors identified four new fungal species originating from the deadwood of different Chinese forest sites. Tohtirjap et al., likewise, present two new fungal species, which were retrieved by linking sequencing techniques with the examinations of trait morphologies. Here, the authors were focusing on the fungal group *Exidia sensu lato*, i.e., fungi of the genera *Exidia, Myxarium*, and *Tremellochaete*, which are known to grow on deadwood in temperate forests worldwide.

In conclusion, the available molecular tools paired with improved assessments of morphological traits and culturing methods are a powerful combination to unravel fungal diversity, taxonomy, and phylogeny. The articles presented in this Research Topic should be viewed as a snapshot of the ongoing and future research on forest mycobiomes and key taxa.

## Author contributions

KG: Writing—original draft, Writing—review & editing.
